# Diarrhœa of Children and Its Treatment

**Published:** 1878-09

**Authors:** 


### The Diarrhea of Children and its Treatment.

After a rapid exposition of the causes and symptoms of infantile diarrhea, M. Blache insist upon the treatment proportioning the medication to the degree of intensity of the disease. As to the question of diet, he recommends regularity of the meals, varying in number according to the age and energy of the child. He insists upon redoubling the vigilance at the time of evolution of the teeth, and especially at the epoch of weaning; the importance of continuing the use of milk in the alimentation of children, and of only abandoning it in persistent diarrhea after having tried the employment of the various kinds of milk, (cow's, goat's and ass's). As a preventive of intestine troubles, M. Blache advises the wearing of a flannel binder, which has the double advantage of protecting the children against chills, and of supporting the belly and preventing that tendency to swelling which occurs on the least intestine trouble. Whatever be the nature of the diarrhea, its origin, its intensity, or the time elapsed since its inception, the treatment which the author proposes, modified according to the cases, has constantly been successful in his hands.

1st. Diminution of the nourishment; appropriate injections repeated as required; and the application of poultices to the belly.

2d. Administer each morning, for three, four or five days in succession, a small teaspoonful of a mixture of *equal parts of castor oil and syrup of acacia*, simply emulsionized by shaking the bottle at the time of giving the medicine.

In order that this medication may produce a curative effect it is necessary to give the mixture in very small doses, and to repeat its employment for several days in succession. Three or four days running is generally sufficient to modify the nature of the stools and to diminish the number. If after two days' treatment the diarrhea is moderated without disappearing it should be suspended for a day or two, and then the same dose should be resumed, but after a days' interval. It is evident that in saying the medium dose of the purgative mixture is a teaspoonful it is understood that the dose must be proportioned according as we may have to do with a child of six weeks or one of eighteen months. To be more precise as to the caster oil, let us say that a gram suffices before six months and 2 or 3 grams up to two years. In cases of an abundant and liquid discharge, repeated twelve or fifteen times, or even more, in twenty-four hours, M. Blache doubles, or even triples the dose of the syrup, and adds to it Sydenham's laudanum (vinum opii) in small quantity, (1 to 3 drops at most in the twenty-four hours, according to the age.) In benign diarrhea the purgative mixture is useless, and the use of chamomile injections for a day or two suffice to entirely arrest the laxity. When to the diarrhea are added manifest signs of gastric derangement, M. Blache does not hesitate in the beginning to administer an ipecac. emetic, and when to a burning fever are joined nervous symptoms, giving rise to a fear of convulsions, he gives calomel in fractional doses before the castor oil—*Jour. de Therap. Can. Jour. Med. Science.—Toledo Med. Journal.*

